# Comment on “Internet-Based Cognitive Behavioral Therapy With Real-Time Therapist Support via Videoconference for Patients With Obsessive-Compulsive Disorder, Panic Disorder, and Social Anxiety Disorder: Pilot Single-Arm Trial”

**DOI:** 10.2196/13234

**Published:** 2020-08-12

**Authors:** David Zargaran, Caoimhe Walsh, Foteini Stefania Koumpa, Muhammad Arsalan Ashraf, Amelia Jayne White, Nikhil Patel, Ravina Tanna, Anna Trepekli, Alexander Zargaran

**Affiliations:** 1 St Thomas' Hospital London United Kingdom; 2 London Deanery London United Kingdom; 3 Oxford Deanery Oxford United Kingdom; 4 Luton and Dunstable Hospital London United Kingdom; 5 Univesity College Hospital London United Kingdom; 6 St George's, University of London London United Kingdom

**Keywords:** internet, CBT, cognitive behavioral therapy, telemedicine, telehealth

We read with great interest the pilot single arm trial by Matsumoto et al [1], which examined the role of internet-based cognitive behavioral therapy (ICBT) via video conference. This paper is both timely and exciting as it assesses the impact of the delivery of remote cognitive behavioral therapy (CBT) to increase access to health care by leveraging improved telecommunication infrastructure.

The authors begin by explaining that difficulties with accessing CBT are due to the expense, number, and uneven urban distribution of therapists, and feel that telemental health has resolved these issues. We would comment that whilst distribution will have been addressed, the use of video conferencing alone would likely be insufficient to address the expense and the paucity of therapists. Therefore, this represents an apparent limitation on the scalability of such interventions. Varying the level of interaction with the therapist through the use of computer-assisted CBT (an empirically developed computer program that builds on CBT skills) has already been described with positive results both in terms of outcomes and cost-effectiveness in a population of primary care patients undergoing treatment for depression [2]. Future work could look to offer a combination of both computer-assisted CBT and video conferencing as a newly derived treatment regimen to assess the efficacy of this combination.

The equivalence of face-to-face CBT and ICBT in a meta-analysis of 1418 patients [3] further strengthens the validity of the conclusions drawn by Matsumoto et al [1]. However, with regards to the methodology employed, the pretrial use of medication duration did not appear to be controlled for, and the article did not state the timing of the interventions. Knowledge of concomitant use of medication could help further delineate the role of ICBT alone in assessing the overall impact. Furthermore, an appreciation of the timing of the CBT sessions could offer therapeutic information as a potential datapoint for the efficacy of CBTs. With specific knowledge of the time an ICBT session took place, one could assess the efficacy of the ICBT session in working hours relative to out-of-hours. Further, the compliance to treatment regimens could be assessed given the increased flexibility of the session appointment times.

Although the single-arm nature of the trial limits the applicability of the conclusions, the authors describe a preference (83% of participants) for video conferencing over face-to-face CBT. More information as to the rationale belying this preference would help inform future intervention strategies. Furthermore, previous experience and perceptions toward face-to-face CBT before an intervention could also help further elucidate the validity of participants’ preference toward video conferencing. One potential explanation could be that the method of participant recruitment and selection could introduce a bias of intrinsic preference of trial subjects toward seeking video-based CBT, given that one recruitment strategy employed by the authors was web-based advertising.

Although a need for further clarification exists, the authors are to be commended on their unique contribution to the field, and it is with great excitement that we anticipate future works to further describe the potential benefits of ICBT and its applications.

## Abbreviations

CBT: cognitive behavioral therapy
ICBT: internet-based cognitive behavioral therapy
